# Adherence to quality breast cancer survivorship care in four Canadian provinces: a CanIMPACT retrospective cohort study

**DOI:** 10.1186/s12885-019-5882-z

**Published:** 2019-07-04

**Authors:** Mary L. McBride, Patti A. Groome, Kathleen Decker, Cynthia Kendell, Li Jiang, Marlo Whitehead, Dongdong Li, Eva Grunfeld, for the CanIMPACT Team

**Affiliations:** 1 Cancer Control Research, BC Cancer, 675 West 10th Avenue, Room 2.107, Vancouver, BC V5Z 1L3 Canada; 2 School of Population and Public Health, University of British Columbia, Vancouver, Canada; 3 Department of Public Health Sciences, Queen’s University, Kingston, Canada; 4 Cancer Research Institute, Queen’s University, Kingston, Canada; 5 Institute of Clinical Evaluative Sciences, Queen’s University, Kingston, Canada; 6 Department of Community Health Sciences, University of Manitoba, Winnipeg, Canada; 7 Epidemiology and Cancer Registry, CancerCare Manitoba, Winnipeg, Canada; 8 Cancer Outcomes Research Program, Dalhousie University and Nova Scotia Health Authority, Halifax, Canada; 9 Critical Care Services Ontario, Toronto, Canada; 10 Department of Family and Community Medicine, University of Toronto, Toronto, Canada; 11 Dalla Lana School of Public Health, University of Toronto, Toronto, Canada; 12 Ontario Institute for Cancer Research, Toronto, ON Canada

**Keywords:** Breast cancer, Breast neoplasms, Primary care, Cancer survivors, Follow-up care, Survivorship care, Guideline-based care, Recommended care, Quality of care, Administrative databases

## Abstract

**Background:**

In order to maximize later health, there are established components and guidelines for quality follow-up care of breast cancer survivors. However, adherence to quality follow-up in Canada may not be optimal, and may vary by province. We determined and compared the proportion of patients in each province who received adherent and non-adherent surveillance for recurrence, new cancers and late effects, recommended preventive care, and recommended physician visits for comorbidities.

**Methods:**

Cohorts consisted of all adult women diagnosed with incident invasive breast cancer between 2007 and 2010/2012 in four Canadian provinces (British Columbia (BC) *N* = 9338; Manitoba *N* = 2688; Ontario *N* = 23,700; Nova Scotia (NS) *N* = 2735), identified from provincial cancer registries, alive and cancer-free at 30 months post-diagnosis. Their healthcare utilization was determined from one to 5 years post-treatment, using linked administrative databases. Adherence, underuse, and overuse of recommended services were evaluated yearly and compared using descriptive statistics.

**Results:**

In all provinces and follow-up years, the majority of survivors had more than the recommended number of visits to either an oncologist or primary care physician (range 53.8% NS Year 3; 85.8% Ontario Year 4). The proportion of patients with the guideline-recommended number of oncologist visits varied by province (range 29.8% BC Year 5; 74.8% Ontario Year 5), and the proportion of patients with less than the recommended number of specified breast cancer-related visits with either an oncologist or primary care physician ranged from 32.6% (Ontario Year 2) to 84.4% (NS Year 3). Underuse of surveillance breast imaging was identified in NS and BC. The proportion of patients receiving imaging for metastatic disease (not recommended in the guidelines) in BC, Manitoba, and Ontario (not reported in NS) ranged from 20.3% (BC Year 5) to 53.3% (Ontario Year 2). Compliance with recommended physician visits for patients with several chronic conditions was high in Ontario and NS. Preventive care was less than optimal in all provinces with available data.

**Conclusions:**

Quality of breast cancer survivor follow-up care varies among provinces. Results point to exploration of factors affecting differences, province-specific opportunities for care improvement, and the value of administrative datasets for health system assessment.

## Background

Breast cancer is the most common cancer in women worldwide. In 2012, nearly 1.7 million women were diagnosed with breast cancer and 6.2 million women had received a prior breast cancer diagnosis [1, 2]. In countries such as Canada with advanced medical care, the five-year survival rate of early-stage breast cancers is 80–90% [1, 2]. However, survivors are at risk for late and ongoing problems including cancer recurrence [3, 4], second cancers [5–7], and physical and cognitive late effects of treatment [8–20], resulting in decreased quality of life [11, 12, 21, 22], higher disability, and mortality [9, 13, 14, 23–25]. In order to optimize cancer survivors’ later health and quality of life, it is critical to deliver comprehensive and appropriate post-treatment care that includes general preventive care, surveillance for recurrences and new cancer, surveillance and management for late effects, and ongoing care for comorbidities [26, 27]. Guidelines for breast cancer-related follow-up care were developed by the American Society for Clinical Oncology (ASCO) [28, 29] and Health Canada [30]. It appears that primary care physicians (PCPs) can provide safe follow-up care for early-stage breast cancer equivalent to oncologists [31], and that they can partner with community specialists in cancer-related follow-up [32], while also managing comorbidities and providing preventive care. However, access and use of guidelines for care, including care for cancer patients, has been variable among United States (US) [33–35] and Canadian physicians [36].

The amount of breast cancer post-treatment care delivered by PCPs differs by province in Canada [37], and underuse and overuse of guideline-based care has been identified in other countries [38, 39] and in two provinces [40] [41, 42]. The objective of this study was to assess and compare quality of post-treatment care in Canadian provinces by determining provincial variation in the level of compliance with guideline-based breast cancer-related survivor care, general preventive care, and ongoing care for comorbidities, using administrative datasets. We quantified and compared the extent of cancer follow-up, chronic and preventive care during survivorship, among four provinces that together represent 58% [43] of the Canadian population.

## Methods

### Setting

The study was conducted in the Canadian provinces of British Columbia (BC), Manitoba (MB), Ontario (ON), and Nova Scotia (NS). In Canada, “medically necessary” healthcare delivery for virtually all residents is the responsibility of provincial/territorial jurisdictions operating according to national legislated healthcare principles of public administration, comprehensiveness, universality, portability, and accessibility [44], resulting in jurisdictional variation in the scope and availability of specific services. Oncology treatment is provided through cancer centres (clinics) and hospitals. Community-based primary care providers (PCPs) and specialist physicians (accessed only through PCP referral) are typically paid using a “fee-for-service” model.

### Design

This work was carried out as part of a Canadian study (Canadian Team to Improve Community-Based Cancer Care along the Continuum: “CanIMPACT”) [45] aimed at improving integration and coordination of care by identifying gaps in care along the breast cancer care trajectory, from diagnosis to survivorship. This investigation tracked the healthcare of retrospective population-based cohorts in each province using linked registries, and clinical and health administrative databases [46]. Detailed descriptions of data sources, linkages, and variables for the full cohorts [37, 46], and the survivorship study cohorts [47] have been previously published; a summary follows. This study was approved by all relevant research ethics boards and data access and privacy committees in each province. Consent was not required. Because of provincial confidentiality requirements, datasets were not able to be combined across provinces. Instead, parallel analyses were conducted in each province.

### Cohort identification and follow-up

The original CanIMPACT cohorts consisted of all women aged 18 years and older diagnosed with incident invasive breast cancer (International Classification of Diseases Version 9: 174.x) from January 1, 2007 identified from each provincial cancer registry. Diagnosis years and follow-up varied according to data availability among provinces. For this study, women diagnosed to the end of 2012 (MB, ON, NS) or 2010 (BC), and alive at 30 months post-cancer diagnosis (to allow for a minimum of 1 year of survivorship care, as patients are censored 6 months before death) were identified. Of this group, women who did not have curative surgery, who had a new primary or recurrence diagnosed in the 27 months after their breast cancer diagnosis, who had metastases identified within 1 year of the breast cancer diagnosis, who did not link to the provincial healthcare insurance plan registries over the entire period from diagnosis date to their end date, or who had less than 1 year of survivorship follow-up were excluded. Women were also excluded if they did not have a valid individual health insurance number or were not residents of their home province at time of diagnosis, had a history of in situ breast cancer or any non-melanoma cancer, or had a histology other than a solid breast cancer [46].

Follow-up was complete to the end of 2013 in ON, 1 October 2013 in NS, end March 2015 in MB, and end 2011 in BC. The post-treatment “survivorship” phase of care was determined to start at 1 year post-diagnosis, continuing for up to 5 years from diagnosis to the end follow-up date, 6 months prior to death date, or 90 days before cancer recurrence or new primary cancer (identified from cancer registry data or billing claims for cancer treatment related to any of local recurrence, regional recurrence and distant metastasis, as indicated by subsequent radiation or surgery or a course of chemotherapy starting more than 2 years after diagnosis).

### Outcomes

Adherence to the ASCO and Canadian cancer follow-up guidelines in effect during the study period [28, 30], guideline-based management of selected co-morbid illnesses, and recommended preventive care outcomes were evaluated. Overuse and underuse were considered. Level of adherence was assessed separately for Years 2–5 of follow-up for all those with full follow-up in that year.

Both ASCO and Canadian guidelines recommend regular physician visits for a medical history and physician examination. Both also advise a regular mammogram, and both recommend against additional tests or imaging in otherwise asymptomatic patients. The ASCO guideline did not recommend that follow-up be with a particular specialist, but did specify “medical oncologists, primary care providers, oncology nurses, (and) surgical oncologists” as some of their target practitioners [28, 29]. The Canadian guideline [30] recommended that responsibility for follow-up be formally allocated to a single physician (without identifying a specialty). All encounters were identified from billing claims records. Since follow-up care in Canada is carried out both by oncologists (defined in our study as radiation, medical, or surgical oncologists, and any surgeon conducting a breast surgery during the follow-up period) and PCPs, we measured visit adherence (number of physician visits) per follow-up year three ways, considering 1) oncologist and PCP visits; (2) oncologist visits only; and (3) breast cancer-related physician visits. For physician visits, adherence was defined as 3 to 4 visits in each of Years 2 and 3; and 2 visits in each of Years 4 and 5. In all provinces except NS, breast mammograms, ultrasounds, and medical resonance imaging (MRI) were classified as surveillance tests if they occurred more than 330 days from the date of the last such test, implying that they were not symptom-related. In NS, these tests were identified as “surveillance” based on procedure codes within the provincial breast screening database. Women with bilateral mastectomy were excluded from counts of surveillance mammograms. In assessing surveillance for recurrence, mammograms, breast ultrasounds and breast MRIs were counted; adherence was defined as one test per follow-up year. Surveillance for metastatic disease was evaluated by counting bone scans, chest imaging with chest x-rays or chest computerized tomographic (CT) scans, abdomen/pelvic imaging with ultrasounds, CT scan, or non-breast-related MRI encounters. Adherence to guidelines for metastatic disease was defined as no surveillance investigations for bone scans, chest imaging with chest x-rays, or chest computerized tomography (CT) scans, abdomen/pelvic imaging with ultrasounds, CT scan, or MRI – all investigations that may be used in the diagnosis of metastatic breast cancer but not recommended for routine surveillance in asymptomatic patients.

Adherence to population-based recommended preventive care and monitoring of common co-morbid chronic illnesses was assessed using published quality indicators [33, 35], and reported as adherent/not adherent. Preventive care assessment included examination of cervical and colon cancer screening, as well as bone densitometry. At least one cervical cancer screen for patients aged 20–69 during the entire follow-up period, with no previous cervical cancer, endometrial and ovarian cancer, and no hysterectomy history, was counted as adherence. Similarly, at least one bone densitometry during follow-up for women aged 65 years or older was considered adherence, and at least one colon cancer screening event for women aged 50–64 during follow-up was considered adherence. For chronic disease management we examined physician visits. Appropriate physician visit frequency for chronic stable angina, congestive heart failure, chronic obstructive pulmonary disease, and diabetes was defined as one visit every 6 months. One visit per year was considered appropriate for those with transient ischemic attack.

### Descriptive variables

To assess comparability of the provincial cohorts, median age at diagnosis (with inter-quartile range), stage at diagnosis (“best stage” recorded in the cancer registries, converted to the TNM 6th edition staging system [48], and treatment received (categorized as lumpectomy, mastectomy, chemotherapy, and adjuvant radiotherapy), were determined. For the 6- to 30-month period prior to diagnosis (baseline), continuity of primary care (measured using the Usual Provider of Care (UPC) Index [49] was calculated. The UPC index was also calculated for PCP and medical oncology care at 1 year after the diagnosis date, at presumed end of primary treatment.

### Statistical analysis

Descriptive statistics were used to characterize each provincial sample. For each provincial follow-up guideline, the frequency and proportion within each adherence category and follow-up year were determined. No statistical comparisons were conducted as these data represent a census experience (no sampling) and study power was high enough that small clinically unimportant differences were likely to be statistically significant.

## Results

There were 9338 survivors from BC, 2688 from MB, 23,700 from ON, and 2735 from NS (Table 1). The shorter median follow-up time in NS was related to an earlier end-of-study date than in other provinces [47]. MB and NS had higher proportions of early-stage (stages I and II) cancers; BC and ON had higher proportions of patients diagnosed with unknown stage. Initial treatment varied among provinces. A lower proportion of BC patients had lumpectomy procedures than the other provinces; a higher proportion of ON patients had mastectomies, a higher proportion of BC and MB patients underwent radiotherapy. An apparently lower proportion of BC patients received chemotherapy, but since BC did not collect chemotherapy data from those women not referred to a cancer centre, there are 14.5% of BC patients with no recorded chemotherapy status, which makes this assessment somewhat inaccurate. In the period prior to diagnosis, 42.3% of BC patients, 47.6% of MB patients, 56.2% of ON patients, and 58.7% of NS patients had high continuity of primary care (UPC). These proportions increased in the survivorship phase for BC and MB, but remained stable for ON and NS (BC 51.4%, MB 57.0%, ON 56.9%, NS 59.2% for NS patients). BC had the highest proportion of patients with no medical oncologist visits in the survivorship phase (57.8% versus a low of 20.6% in ON) and a corresponding low proportion of patients with high medical oncology continuity of care (16.8% versus a high of 38.9% in ON).

**Table 1:** Characteristics of Provincial Cohorts of Breast Cancer Survivors

Variable	BC	MB	ON	NS
Overall N	9338	2688	23,700	2735
Diagnosis years	2007–2010	2007–2012	2007–2012	2007–2012
Follow-up Time (N,%)				
Median (Inter-quartile range)	4 (3–4)	4 (3–4)	5 (5–5)	2 (2–4)
Full follow-up in Year 2	9338 (100.0)	2688 (100.0)	23,700 (100.0)	2735 (100.0)
Full follow-up in Year 3	8862 (94.9)	2583 (96.1)	22,297 (94.1)	2064 (75.5)
Full follow-up in Year 4	6213 (66.5)	2037 (75.8)	21,148 (89.2)	1726 (63.1)
Full follow-up in Year 5	3865 (41.4)	1516 (56.4)	17,255 (72.8)	892 (32.6)
Age at Diagnosis, years				
Median(Inter-quartile range)	60(51–70)	61(51–70)	60 (50–69)	61 (51–70)
Stage at Diagnosis (N,%)				
I	4244 (44.7)	1268 (47.2)	10,036 (42.3)	1366 (50.0)
II	3150 (33.2)	1086 (40.4)	8602 (36.3)	1010 (36.9)
III	1089 (11.5)	317 (11.8)	2660 (11.2)	315 (11.5)
IV	182 (2.0)	7 (0.3)	88 (0.4)	23 (0.8)
Unknown	828 (8.7)	10 (0.4)	2314 (9.8)	21 (0.8)
Initial Treatment (N,%)				
Lumpectomy				
Yes	4890 (51.5)	1948 (72.5)	17,275 (72.9)	1970 (72.0)
No	4603 (48.5)	740 (27.5)	6425 (27.1)	765 (28.0)
Mastectomy				
Yes	3535 (37.2)	910 (33.9)	8293 (35.0)	1360 (49.7)
No	5958 (62.8)	1778 (66.1)	15,407 (65.0)	1375 (50.3)
Chemotherapy				
Yes	3391 (35.7)	1244 (46.3)	10,884 (45.9)	1151 (42.1)
No	4724 (49.8)	1444 (53.7)	12,816 (54.1)	1584 (57.9)
Unknown	1378 (14.5)	0	0	0
Radiotherapy				
Yes	6079 (64.0)	1550 (57.7)	15,318 (64.6)	1559 (57.0)
No	3414 (36.0)	1138 (42.3)	8382 (35.4)	1176 (43.0)
Baseline Continuity of Care (N,%)				
0 visit	701 (7.4)	133 (5.0)	1448 (6.1)	93 (3.4)
1–2 visits	1175 (12.4)	229 (8.5)	2514 (10.6)	333 (12.2)
UPC < =0.75(Low)	3605 (38.0)	1046 (38.9)	6414 (27.1)	703 25.7)
UPC > 0.75(High)	4012 (42.3)	1280 (47.6)	13,324 (56.2)	1606 (58.7)
Survivorship PCP continuity of care (N,%)				
0 visit	395 (4.2)	84 (3.3)	624 (2.8)	115 (4.2)
1–2 visits	926 (9.8)	145 (5.6)	1470 (6.6)	413 (15.1)
UPC < =0.75(Low)	3289 (34.7)	883 (34.2)	7513 (33.7)	589 (21.5)
UPC > 0.75(High)	4883 (51.4)	1471 (57.0)	12,690 (56.9)	1618 (59.2)
Survivorship Medical Oncologist continuity of care (N,%)				
0 visit	5494 (57.8)	923 (35.7)	4603 (20.6)	829 (30.3)
1–2 visits	1468 (15.5)	621 (24.0)	5066 (22.7)	1250 (45.7)
UPC < =0.75(Low)	945 (10.0)	345 (13.4)	3960 (17.8)	71 (2.6)
UPC > 0.75(High)	1592 (16.8)	694 (26.9)	8668 (38.9)	585 (21.4)

*PCP* Primary Care Provider

*UPC* Usual Provider Continuity index

### Adherence to guideline-based care

**Fig. 1:**
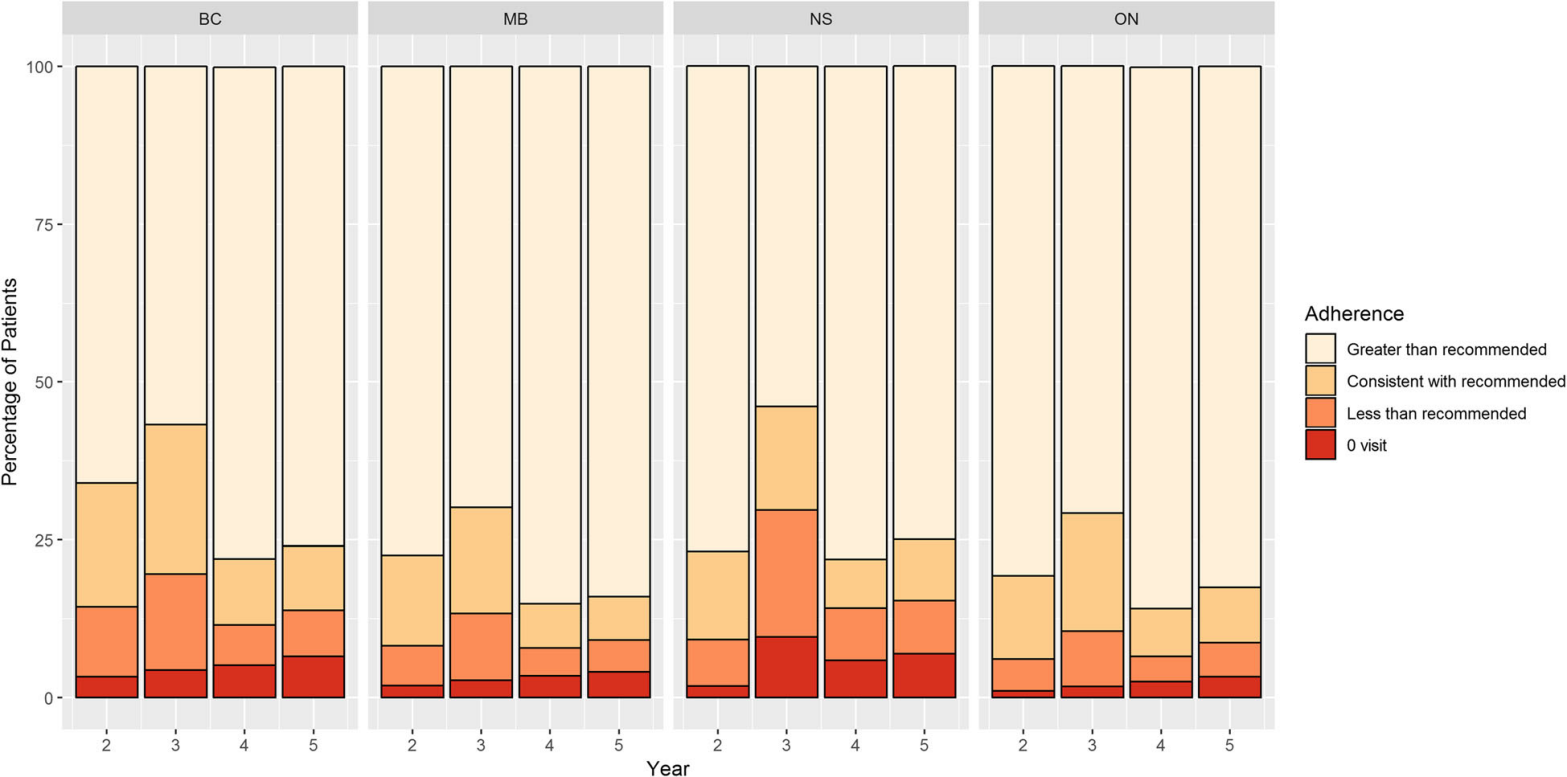
Visit Adherence (PCP or Oncology)

Oncologist or primary care physician visits: For all provinces and in all follow-up years, the majority of survivors had more than the recommended number of patient visits to oncologists or PCPs (range 53.8% in NS Year 3; 85.8% in ON Year 4) (Fig. 1). The proportion of patients with fewer than the recommended number of visits (including 0 visits) ranged from 6.1% (ON Year 2) to 29.7% (NS Year 3).

**Fig. 2:**
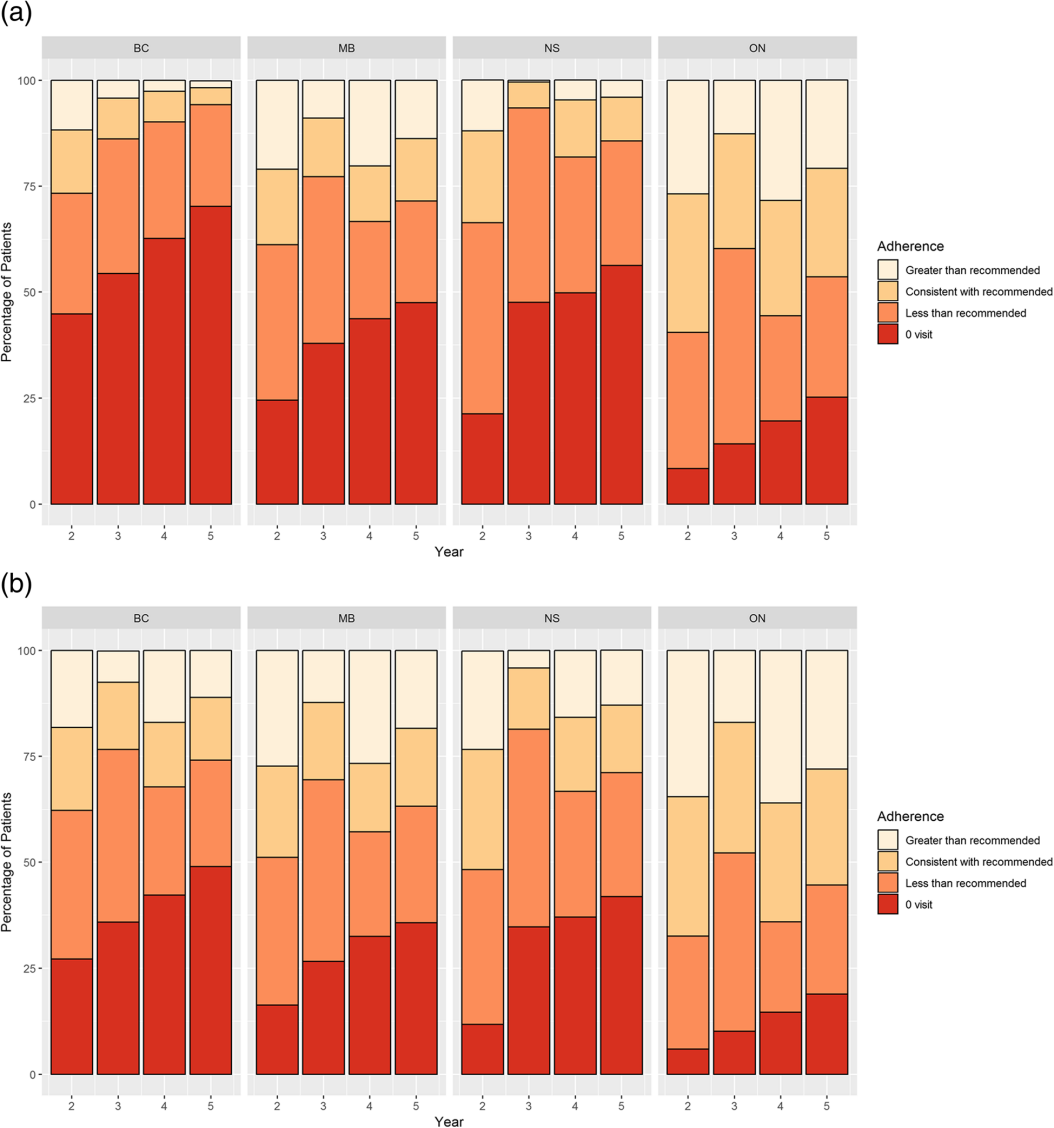
**a** Oncologist Visit Adherence. **b** Breast cancer-related PCP or Oncology Adherence

Oncologist visits: Considerable inter-provincial variation in the proportion of survivors visiting an oncologist was observed, although all provinces showed a decrease in the proportion of survivors without an oncologist visit over time (Fig. 2a). In each follow-up year, BC had the lowest proportion of survivors with any oncologist visit (56.2% Year 2; 29.8% Year 5). MB and NS had similar oncologist visit rates (MB 75.5% Year 2, 52.5% Year 5; NS 78.7% Year 2, 43.7% Year 5). ON had the highest proportion of survivors who saw an oncologist annually (91.7% Year 2, 74.8% Year 5).

Breast cancer-related oncologist or primary care physician visits: When breast cancer was reported as the reason for visit, a much smaller proportion of patients had more than the recommended number of patient visits (range 4.1% in NS Year 3; 36.0% in ON Year 4) (Fig. 2b). There were similar proportions of recommended, less than recommended, and greater than recommended visits in BC, MB, and NS. ON had the highest proportion of visits consistent with recommendations (range 32.9% Year 1 to 27.4% Year 4).

**Fig. 3:**
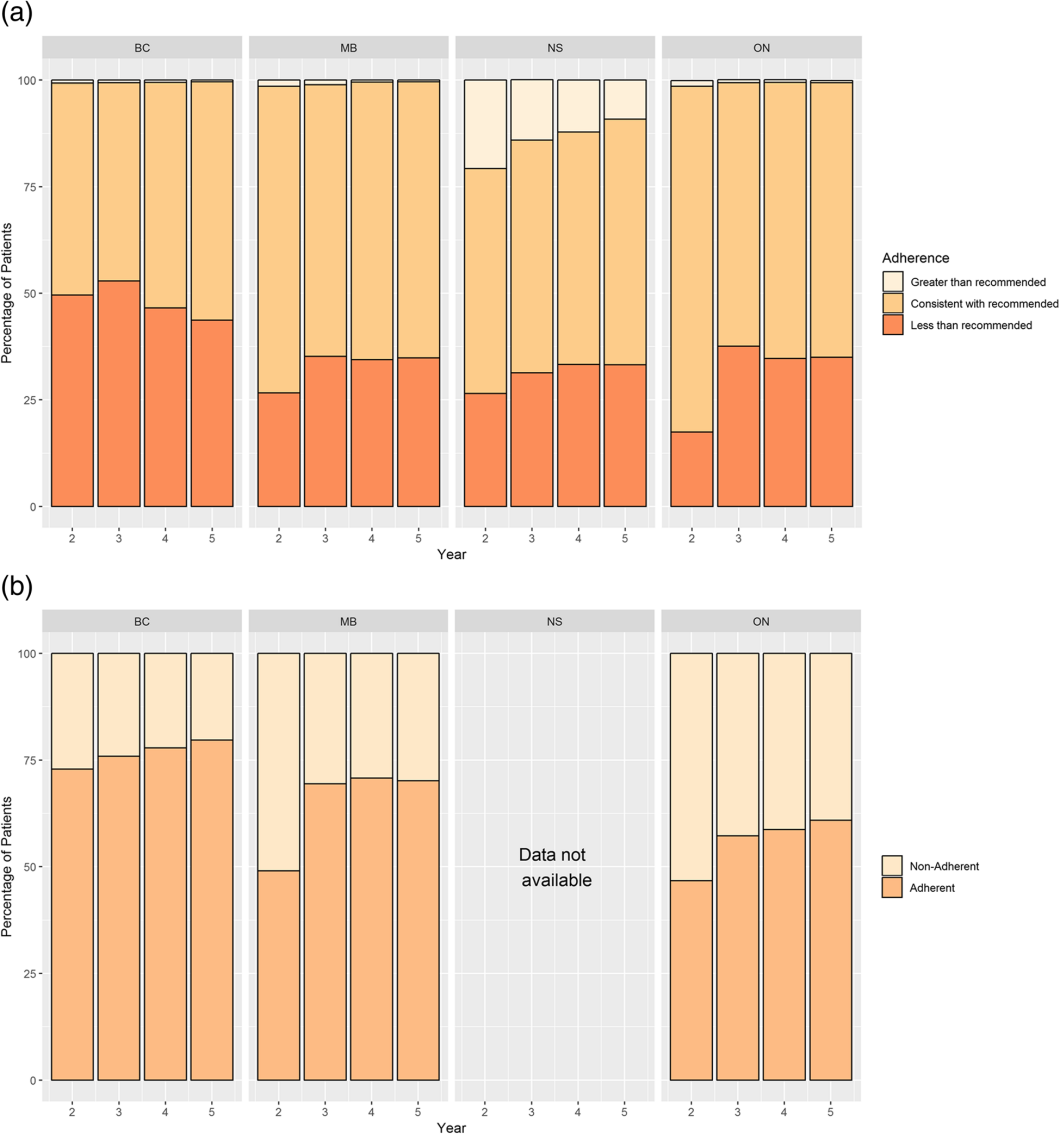
**a** Surveillance Breast Imaging. **b** Imaging for Metastatic Disease

Surveillance breast imaging: For all provinces, the majority of survivors in each follow-up year received guideline-based surveillance imaging (Fig. 3a), with MB and ON reporting higher adherence than BC and NS. A greater proportion of survivors in NS received surveillance imaging in excess of guideline recommendations (between 9.1 and 20.7%) compared to other provinces. In BC, a greater proportion of survivors received fewer imaging examinations than supported in the guidelines (between 43.7 and 50.2%) compared to other provinces.

Imaging for metastatic disease: Routine imaging for metastatic disease is not recommended in surveillance of asymptomatic breast cancer patients. Between 20.3% (BC, Year 5) and 53.3% (ON, Year 2) of survivors did undergo imaging for metastatic cancer in the follow-up period (Fig. 3b). Considerable variation between provinces was observed, with adherence to this recommendation highest in BC, and lowest in ON (NS data not available).

### Chronic disease management

Prevalence of chronic stable angina (0.5 to 3.2% of provincial cohorts), congestive heart failure (1.0 to 1.6%), chronic obstructive pulmonary disease (2.0 to 3.0%), transient ischemic attacks (0.4 to 0.8%), and diabetes (9.8 to 11.5%) was determined (Table 2). Small numbers meant that compliance with some guidelines was not measured in MB. High levels of compliance with physician visit recommendations for care of patients with any of these conditions were seen in ON (range 92.7–99.2%) and NS (97.1–100%), suggesting that these comorbidities were well managed among patients in these provinces. Much lower levels of compliance (range 6.0–30.6%) were observed in BC.

**Table 2:** Prevalence and adherence for chronic diseases and preventive care

	BC	MB	ON	NS
Overall *N* with chronic disease and/or preventive care	8862	2583	22,297	2735
Diagnosis years	2007–2010	2007–2011	2007–2010	2007–2012
Prevalence for Chronic Disease (N, %)				
Chronic Stable Angina (CSA)	283 (3.2)	24 (0.9)	572 (2.6)	14 (0.5)
Congestive Heart Failure (CHF)	143 (1.6)	26 (1.0)	239 (1.1)	35 (1.3)
Chronic Obstructive Pulmonary Disease (COPD)	228 (2.6)	53 (2.1)	437 (2.0)	82 (3.0)
Transient Ischemic Attack (TIA)	55 (0.6)	21 (0.8)	124 (0.6)	11 (0.4)
Diabetes (DM)	1022 (11.5)	255 (9.9)	2189 (9.8)	310 (11.3)
Adherence to Chronic Disease Indicators (%)				
CSA: 6-month Visits	6.0	–	95.6	100
CHF: 6-month Visits	23.8	–	92.9	97.1
COPD: 6-month Visits	9.2	–	92.7	98.8
TIA: 1-year Visits	14.5	–	99.2	100
DM: 6-month Visits	30.6	99.6	95.8	97.4
Prevalence for Preventive Care (N, %)				
Cervical cancer screening	6573 (69.2)	–	14,934 (67.0)	2036 (74.7)
Bone densitometry	2705 (28.5)	–	8625 (38.7)	–
Colorectal cancer screening	4833 (50.9)	–	10,233 (45.9)	–

### Preventive care

Cervical cancer screening was recorded in 69.2% (BC), 67.0% (ON), and 74.7%% (NS) of eligible women (Table 2; MB data not available). Bone densitometry was reported for 28.5% of women in BC, and 38.7% of those in ON (NS and MB data not available). Finally, 50.9% of age-eligible women in BC had colon cancer screening tests, and 45.9% of eligible BC women (NS and MB data not available).

## Discussion

We conducted a population-based retrospective cohort study of four Canadian provinces that examined and compared the quality of care for breast cancer survivors within a publicly-funded, comprehensive healthcare framework covering virtually all residents. We found considerable provincial variation in levels of reported guideline-based follow-up care, chronic disease management, and preventive care. These differences suggest that there may be both overuse and gaps in care at different points in the system and to a variable extent among provinces, which could indicate province-specific opportunities for care improvement. For instance, differences in continuity of medical oncologist visits may be related to differences in provincial organization of cancer care and cancer discharge policies. The results for oncologist follow-up visits are consistent with the fact that BC supports early discharge while ON tends to retain cancer patients longer in its cancer centres [47]. The resulting difference in type of follow-up physician may, in part, account for the observed variation in adherence to guideline-based surveillance imaging and preventive care. The results also suggest there may be access issues such as physician ability to obtain guidelines [36], and geographic availability of PCPs, oncologists, and services such as imaging equipment. Lack of a PCP is a particular concern. In 2016, approximately 12% of Canadian women aged 12 and older reported that they did not have a regular medical doctor or other healthcare provider [50]. The small amount of overuse of surveillance imaging in BC, MB and ON (Fig. 3a) may be a result of too restrictive definition of an eligible test in our study (imaging only considered “surveillance” if > 330 days from last such test), as other ON studies reported multiple surveillance tests in Year 2 post-treatment [41, 42]. In NS, a different approach was utilized based on availability of screening imaging event data in the provincial screening database, which would account for the higher proportion of patients reported to have received surveillance imaging in excess of guideline recommendations.

Similar to one Dutch study that included all adult-aged women [51], but in contrast to another Dutch study measuring hospital follow-up [52], in our study more women had more than the recommended number of visits to either oncologists or PCPs. However, the trend over time differed. In the Dutch study [51], women had less than the recommended frequency of surveillance mammography; among our study patients, receipt of surveillance mammography was generally consistent with guidelines, but a sizeable minority also had fewer than recommended surveillance mammograms.

Our results may also be affected by jurisdictional differences and misclassification in identifying patient eligibility, completeness of data collection, or incomplete or biased capture of events due to limitations in the definition of outcomes or differences in data sources. For instance, end of primary treatment date was not available in all provinces, so could not be used as the definition of the start of follow-up. We chose a date that we felt would provide high confidence that all patients meeting the survivor criteria (including undergoing primary surgery, with no recorded new primary cancer, recurrence, or metastases at 1 year post-diagnosis) would be in the follow-up phase of care. MRI encounters were not captured in BC since MRI facilities are facility-funded, rather than fee-for-service. In our study, a yearly MRI or ultrasound on its own was considered as adherence to surveillance. However, a common clinical scenario is a surveillance mammogram that identifies something suspicious and recommends more imaging (perhaps by MRI or ultrasound) which then resolves the issue as being benign. Counting those imaging events is probably not appropriate, since they are not surveillance-based; however, since indications for imaging were not available, these could not be excluded. Similarly, the differences in adherence to chronic disease care in BC may be a result of incomplete recording of these activities as reasons for visits. The decrease in overuse of surveillance imaging from Year 2 to Year 5 in NS compared to other provinces may be an artefact of coding quality issues in earlier years of the NS Breast Cancer Screening Program database rather than clinical practice.

The findings presented in this study are based on outpatient healthcare administrative data over several years among geographically-defined populations in several jurisdictions with the same healthcare framework but differences in approaches to healthcare delivery. Administrative data can provide more accurate estimates of medical care than alternative approaches based on more indirect data sources, and have been used to examine follow-up care among older breast cancer survivors in the US [53] and those within a large integrated healthcare system [54]. The comparative analysis of these data permits direct assessment of the relative performance across provinces in delivery of guideline-based care at the population level in the first 5 years post-treatment, when surveillance is most intensive [55]. Although results are generated from the Canadian health system, they do provide real-world data in a total geographically-defined adult breast cancer population, compared to US Medicare, which includes only women 65 years and over plus some at-risk subgroups; and healthcare systems that do not cover entire population of a specific area. However, there are some limitations to the study. Follow-up time and case retention varied among the provinces, introducing some error. Data comparability was affected due to provincial differences in the scope of care provided and recording of events. For some surveillance measures, we could not always separate out tests that may have been for symptoms or new findings on physical examination. Importantly, these results do not provide any direct information about the reasons for the observed differences in care. In order to inform care improvement, an assessment of sociodemographic, clinical, healthcare delivery, and system factors that drive these differences, is needed. In addition, the perspectives of healthcare managers, oncologists, FPs and survivors about barriers and facilitators of quality care uptake are required, to guide future strategies and interventions to improve care delivery. Lastly, there are additional challenges of data availability, completeness, comparability and quality that make such comparative studies more challenging across different healthcare systems [55, 56].

## Conclusions

This study found deficiencies and inefficiencies in follow-up care for breast cancer survivors, and differences between provinces within the Canadian healthcare system. Further study is needed to identify modifiable factors in order to improve follow-up care of breast cancer survivors by primary care providers. This investigation demonstrates the value of registries and healthcare administrative datasets in assessing the quality of healthcare. However, further work is needed to improve comparability of such data across jurisdictions.

## Data Availability

The data supporting the conclusions of this article are not available in a public repository, in accordance with provincial government policies. They are housed at Population Data BC (BC), Manitoba Population Research Data Repository (MB), Institute for Clinical Evaluative Sciences (ON), and Health Data Nova Scotia (NS).

## Abbreviations

ASCO: American Society for Clinical Oncology
BC: British Columbia
CanIMPACT: Canadian Team to Improve Community-Based Cancer Care along the Continuum
CHF: Congestive Heart Failure
COPD: Chronic Obstructive Pulmonary Disease
CSA: Chronic Stable Angina
CT: Computerized tomography
DM: Diabetes
MB: Manitoba
MRI: Medical resonance imaging
NS: Nova Scotia
ON: Ontario
PCP: Primary care physicians
TIA: Transient Ischemic Attack
UPC: Usual Provider of Care
US: United States
